# Pseudoaneurysm of the uterine artery with presentation of haematuria; a rare case report

**DOI:** 10.1016/j.ijscr.2021.106675

**Published:** 2021-12-09

**Authors:** Majid Anwer, Anil Kumar, Anurag Kumar, Subhash Kumar, Deepak Kumar, Farheen Ahmed

**Affiliations:** a Department of Trauma & Emergency (Gen Surgery), All India Institute of Medical Sciences, Patna, India; b Department of Radio Diagnosis, All India Institute of Medical Sciences, Patna, India; c Department of General Surgery, All India Institute of Medical Sciences, Patna, India; d Department of Anaesthesiology, All India Institute of Medical Sciences, Patna, India

**Keywords:** Uterine artery pseudoaneurysm, Haematuria, Digital subtraction angiography (DSA), Angioembolization, .

## Abstract

**Introduction:**

Pseudoaneurysm of the uterine artery is a condition in which extra luminal collection of blood with a turbulent flow that communicates with flowing blood of uterine artery through a defect in its arterial wall. As per literature uterine artery pseudoaneurysm is a very rare condition and its incidence is 2–3/1000 deliveries. Clinical diagnosis is very challenging and in the index case haematuria was the presenting complaint which in fact is the extremely rare presenting complaint. Angioembolization is the ideal treatment modality for such a rare condition.

**Case presentation:**

A 25-year old female presented in a shock state with history of massive haematuria two months after delivering a baby. She was resuscitated with fluid, blood and blood products. A computed tomography angiogram was done which showed a large pseudoaneurysm of the left uterine artery so consequently angioembolization was done with n-butyl cyanoacrylate (NBCA) and lipoid mixture. Serial assessment of biochemical and clinical parameters depicted improvement in the clinical status of the patient. She was doing well at 6 months of follow up.

**Discussion:**

A post-partum massive haematuria could be due to pseudo aneurysm of uterine artery. The presentation of haematuria may occur due to communication of aneurysm with urinary bladder and which further get ruptured. Aggressive resuscitation and angioembolization of the pseudoaneurysm is employed to treat such patients.

**Conclusion:**

Pseudoaneurysm of uterine artery is rare condition which may present as haematuria. Once clinical diagnosis is suspected it's better to first resuscitate and plan for angioembolization for better outcome.

## Highlights

• Pseudoaneurysm of uterine artery presented with haematuria is the first case to be reported in the leterature.
• Clinically the pseudoaneurysm of uterine artery should be considered in mind in patients with massive haematuria after caesarean/normal deliveries.
• Aggressive resuscitation and definitive management with embolization is the ideal treatment for such cases.

## 1 Introduction

Pseudoaneurysm of the uterine artery (PUA) is a condition in which extra luminal collection of blood with a turbulent flow that communicates with flowing blood of uterine artery through a defect in its arterial wall. The uterine artery pseudoaneurysm is a very rare condition and its incidence is two to three per thousand deliveries (2–3/1000). This condition is an unusual complication after the normal vaginal delivery, caesarean section, myomectomy, or hysterectomy [1]. The usual presentation for PUA is bleeding per vagina after rupture of pseudoaneurysm [2]. Because of this haemorrhagic presentation this condition is life threatening and require urgent diagnosis on high degree of clinical suspicion [3], [4]. Initial evaluation may be done by ultrasonography but diagnostic confirmation is made on computed tomography and arteriography [5], [6]. In the diagnostic step, trans catheter uterine artery embolization (UAE) has considered as an appropriate treatment for the pseudoaneurysm of uterine artery as in index case [7]. We report here a case of pseudoaneurysm of the uterine artery presenting with rare complaint of massive haematuria after caesarean section which was managed successfully with embolization. Such presentation of massive haematuria in PUA is the first case as per my best knowledge. This case report is in line with SCARE 2020 criteria [8].

## 2 Case presentation

**Fig. 1:**
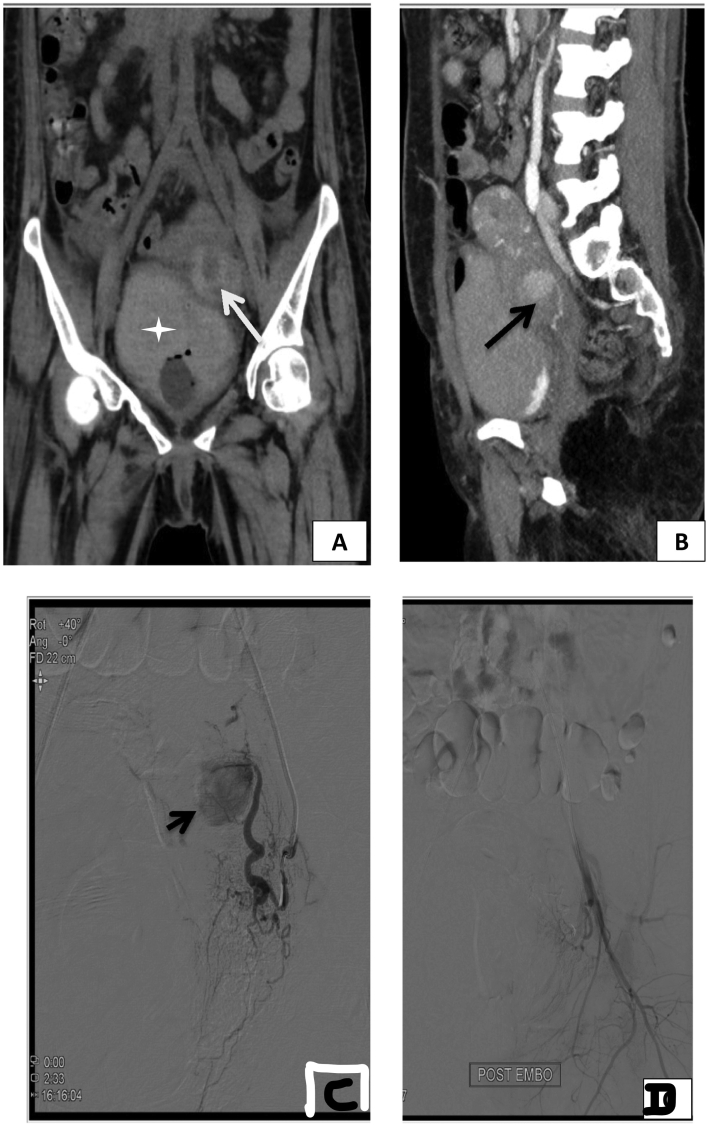
Images of uterine artery pseudoaneurysm. A: Coronal unenhanced CT image showing hyper dense clot in the urinary bladder (white star) and hyper dense haematoma in the pelvis on left side (white arrow). B: Sagittal reformatted maximum intensity projection image of CT angiography, showing a pseudoaneurysm between the bladder and the lower uterus with a tortuous artery reaching up to it (black arrow). C: Digital subtraction angiography image of super selective left uterine artery injection showing the pseudoaneurysm from the distal uterine artery. D: Post-embolization image of left internal iliac artery injection showing non-opacification of the pseudoaneurysm.

A 25 year old lady presented in a state of shock with massive haematuria. Patient gave a previous history of lower segment caesarean section 2 month ago following which she was doing well for one month. On 30th post-operative day of caesarean, she started passing blood and blood clots (long cylinder) in urine. There was no history of bleeding from per vaginum and other site. There was no history of dysuria or trauma and also no significant history of drugs or allergy found. She was evaluated outside and a CT was done which suggested a uterine/internal iliac artery pseudoaneurysm along with bladder clots. A cystoscopy was done outside 4 days before presentation which showed a defect of size 2.2 cm on dome of bladder. Intrabladder blood clot was evacuated. She was then referred to our center. On arrival she was haemodynamically unstable and her pulse rate, blood pressure, haemoglobin, blood urea, and serum creatinine were 154/min, 62/40 mm Hg, 4.8 g/dl, 10.8 mg/dl, and 0.44 mg/dl respectively. She was aggressively resuscitated with crystalloids and later 4 PRBC and 4 FFP was transfused. She was started on inotropic support and shifted to ICU for stabilisation. She responded to fluid resuscitation and her BP and pulse normalised. She developed blockage of the Foley catheter with clot retention in bladder. Foley catheter was removed and a tri-way Foley catheter with bladder irrigation was started. Later urine got cleared after irrigation. After 20 h when the patient stabilised, a CT angiography was done to localise the source of bleeding. CT angiogram was suggestive of left uterine pseudoaneurysm. She was taken to DSA after stabilisation which showed a pseudoaneurysm in the pelvis on the left side between the bladder and the lower uterus, possibly from the anterior uterine wall, with surrounding haematoma. There was clot and air within the urinary bladder; however no direct vesico-vaginal or vesico-uterine fistula was evident (Fig. 1A and B).The patient was urgently taken up for digital subtraction angiography and subsequent embolization. It revealed a hypertrophied and tortuous left uterine artery with a large pseudoaneurysm distally. Then after super selective microcatheterisation using 2.7 F microcatheter of left uterine artery, successful glue embolization was performed using 20% n-butyl cyanoacrylate and lipiodol mixture (Fig. 1C). Post procedure control angiogram showed complete devascularisation of pseudoaneurysm (Fig. 1D). This intervention was done by an additional professor with 12 years of experience in Level-1 trauma center. She was remaining haemodynamically stable throughout the procedure. In the post angio period she was kept in the ICU for 2 days where her condition remains stable. Haematuria persisted for 2 days and then it gradually cleared. She was later shifted to general wards from where she was discharged. She was doing well at 6 months of follow up.

## 3 Discussion

The vascular abnormality from vascular damage leads to disruption and defect within the arterial wall. Blood flow in such a damaged artery usually dissects the adjacent tissue, and forming a sac which communicates with the arterial lumen. Histologically pseudoaneurysm differentiated from true aneurysm by lacking the three-layered wall unlike true aneurysm [9].Uterine artery pseudoaneurysm is an uncommon complication after caesarean section. Yosuke et al. compiled a list of 29 cases of pseudoaneurysm [10]. The most common presentation with such pseudoaneurysm is asymptomatic or delayed post-partum haemorrhage with bleeding per vaginum [11], [12], [13], [14]. Our case is unique in the way that the patient presented in a state of shock with massive haematuria. To our knowledge this is the first case where the presentation was unique with massive haematuria. In the index case, the false aneurysm made after communication with the urinary bladder and patient here presented with massive haematuria. The possible mechanism could have been the rupture of pseudoaneurysm in the bladder as it was in close proximity. Pseudoaneurysm of the uterine artery can occur due to normal vaginal delivery, caesarean section, myomectomy, or hysterectomy [1], [2]. There are several diagnostic approaches including ultrasonography, CT, and magnetic resonance imaging to identify the pseudoaneurysm of the uterine artery [5], [6], [15]. Initially, such conditions were treated by exploratory laparotomy, and internal iliac artery ligation [16]. The case was successfully managed with aggressive resuscitation and later digital subtraction angiography and embolization of the uterine artery. Presently, image guided catheter embolization has become the standard method of treatment like our case [9], [17]. Other possible treatment options are US guided thrombin injection or covered stent depending on the expertise of the interventionist [18], [19]. Like present case there are few cases reported in literature which were also managed by embolization [20], [21], [22].

## 4 Conclusion

Post-partum massive haematuria could be due to pseudoaneurysm of uterine artery. A high index of suspicion for pseudoaneurysm of uterine artery must be considered in mind in patients with massive haematuria after caesarean/normal deliveries. Aggressive resuscitation and definitive management with embolization is the ideal treatment for such cases.

## Patient's perspective

I am so grateful to the trauma team who managed me without operative intervention, as I was very fearful even to think about surgery and anaesthesia.

## Consent

Written informed consent was obtained from the patient for publication of this case report and accompanying images. A copy of the written consent is available for review by the Editor-in-Chief of this journal on request.

## Provenance and peer review

Not commissioned, externally peer-reviewed.

## Ethical approval

N/a.

## Funding

None.

## Guarantor

Dr Anil Kumar.

## Research registration number

N/a.

## CRediT authorship contribution statement

Majid Anwer: Study concept
Anil Kumar: Writing the paper.
Anurag Kumar: Data collection
Subhash Kumar: Radiological intervention (embolization)
Deepak Kumar: Data analysis
Farheen Ahmed: References and drafting the manuscript.

## Declaration of competing interest

None declared.
